# Diffusion tensor imaging in children with unilateral hearing loss: a pilot study

**DOI:** 10.3389/fnsys.2014.00087

**Published:** 2014-05-26

**Authors:** Tara Rachakonda, Joshua S. Shimony, Rebecca S. Coalson, Judith E. C. Lieu

**Affiliations:** ^1^Department of Otolaryngology-Head and Neck Surgery, Washington University School of MedicineSt. Louis, MO, USA; ^2^Department of Radiology, Mallinckrodt Institute of Radiology, Washington University School of MedicineSt. Louis, MO, USA; ^3^Departments of Neurology and Radiology, Washington University School of MedicineSt. Louis, MO, USA

**Keywords:** hearing loss, unilateral hearing loss, diffusion tensor imaging, children, magnetic resonance imaging

## Abstract

**Objective**: Language acquisition was assumed to proceed normally in children with unilateral hearing loss (UHL) since they have one functioning ear. However, children with UHL score poorly on speech-language tests and have higher rates of educational problems compared to normal hearing (NH) peers. Diffusion tensor imaging (DTI) is an imaging modality used to measure microstructural integrity of brain white matter. The purpose of this pilot study was to investigate differences in fractional anisotropy (FA) and mean diffusivity (MD) in hearing- and non-hearing-related structures in the brain between children with UHL and their NH siblings.

**Study Design**: Prospective observational cohort.

**Setting**: Academic medical center.

**Subjects and Methods**: Sixty one children were recruited, tested and imaged. Twenty nine children with severe-to-profound UHL were compared to 20 siblings with NH using IQ and oral language testing, and MRI with DTI. Twelve children had inadequate MRI data. Parents provided demographic data and indicated whether children had a need for an individualized educational program (IEP) or speech therapy (ST). DTI parameters were measured in auditory and non-auditory regions of interest (ROIs). Between-group comparisons were evaluated with non-parametric tests.

**Results**: Lower FA of left lateral lemniscus was observed for children with UHL compared to their NH siblings, as well as trends toward differences in other auditory and non-auditory regions. Correlation analyses showed associations between several DTI parameters and outcomes in children with UHL. Regression analyses revealed relationships between educational outcome variables and several DTI parameters, which may provide clinically useful information for guidance of speech therapy.

**Discussion/Conclusion**: Our data suggests that white matter microstructural patterns in several brain regions are preserved despite unilateral rather than bilateral auditory input which contrasts with findings in patients with bilateral hearing loss.

## Introduction

Although unilateral hearing loss (UHL) often goes undetected until children begin school, prevalence rates of UHL in newborns range from 0.04 to 3.4% (Mehl and Thomson, 1998; Widen et al., 2000). Studies have estimated the prevalence of UHL in school-aged children (ages 6–19 years) as high as 5% (Niskar et al., 1998). It was previously assumed that because children with UHL had one functioning ear, speech acquisition and language comprehension skills developed normally. However, several studies have suggested otherwise (Davis et al., 1981; Bess and Tharpe, 1984; Lieu et al., 2010). In one study, when compared with their normal hearing (NH) siblings, children (ages 6–12 years) with UHL had substantially worse oral language scores (Lieu et al., 2010). Other investigators have found evidence of increased rates of behavioral problems, a greater need for intensive educational plans and worse performance in school amongst children with UHL (Bess, 1982; Bess and Tharpe, 1984; Davis et al., 2002).

Presumably, problems with sound localization and the need for a higher signal-to-noise ratio for speech comprehension in children with UHL contribute to their delay in language skills, but they do not explain why some of these children experience behavioral challenges (Bess and Tharpe, 1984; Davis et al., 2002). These difficulties may reflect problems with executive functioning rather than difficulty with auditory processing, similar to children with bilateral hearing loss who may encounter executive functioning difficulties (Figueras et al., 2008; Beer et al., 2011).

Whether “right ear advantage (REA)” exists for speech perception has been controversial (Hugdahl, 2011). Although children with UHL have performed worse on speech-language tests, it is less clear that the side of hearing impairment influences cognitive abilities. According to the REA hypothesis, children with right UHL should have greater difficulties with language skills than those with left UHL. Some have postulated that the right hemisphere preferentially processes spectrally complex sounds (e.g., music), whereas the left hemisphere processes temporally complex sounds (e.g., speech) (Penhune et al., 1996). Older studies point to a higher rate of grade failures and worse verbal test performance in children with right UHL (Bess and Tharpe, 1984; Hartvig et al., 1989; Niedzielski et al., 2006). However, a large case-control study did not find intellectual differences based upon side of hearing impairment (Lieu et al., 2010).

In this study, we sought to find a neuroanatomical basis for educational differences. In a prior investigation in this study cohort, differences in resting state functional connectivity MRI (rs-fcMRI) were found in areas associated with auditory processing, executive function and memory formation between children with UHL and NH controls (Tibbetts et al., 2011). MRI provides a non-invasive means by which to examine the brain for gross anatomical changes, functional changes in activation (fMRI), and microstructural changes (diffusion tensor imaging, DTI) in numerous nervous system pathologies (Rykhlevskaia et al., 2008), and more specifically in hearing loss (Chang et al., 2004; Firszt et al., 2006; Lin et al., 2008; Kim et al., 2009; Propst et al., 2010). DTI measures of microstructural damage in white matter have been associated with behavioral measures in numerous diseases, such as in blindness (Shimony et al., 2006), depression (Shimony et al., 2009), traumatic brain injury (Hulkower et al., 2013), phenylketonuria (Antenor-Dorsey et al., 2013), and numerous other examples. DTI measures the diffusion of water molecules in brain tissues (Shimony et al., 2006). Water in brain tissues diffuses faster parallel to white matter tracts as compared to perpendicular, a property known as anisotropy. Anisotropy has been used to investigate the integrity and course of white matter tracts in the brain. Common DTI parameters include fractional anisotropy (FA), which ranges from 0 for equal diffusion in all directions to 1 for diffusion only along 1 axis, and the mean diffusivity (MD), which measures how easily water diffuses averaged over all directions, also ranging from 0 to 1. Changes in diffusivity or loss of directionality suggest lack of microstructural integrity (Mori and Zhang, 2006; Assaf and Pasternak, 2008; Neil, 2008). Restricted diffusion has been identified in several types of disease processes, including acute demyelination, certain brain tumors and acute ischemia (Mukherjee et al., 2008).

A few studies have used DTI to investigate the neuroanatomic properties of auditory processing regions in patients with hearing loss. These studies focused mainly upon subcortical structures and adults with hearing loss (Chang et al., 2004; Lutz et al., 2007; Lin et al., 2008; Kim et al., 2009; Wu et al., 2009). In light of DTI changes reported in prior studies of hearing loss, the goal of this study was to determine how UHL influences the development of white matter tracts in the brain. Our aims were to compare the microstructural integrity of white matter tracts using DTI in hearing-related and non-hearing-related brain regions between children with UHL and their NH siblings; and to examine relationships between various educational outcome variables and the DTI parameters (FA and MD) of hearing-related regions.

## Materials and methods

### Study participants

Sixty one participants with severe-to-profound UHL and their NH siblings were enrolled. Participants with UHL were defined as having severe-to profound sensorineural UHL by pure tone audiometry, with pure tone averages (PTA) ≥70 dB hearing level (HL) in the affected ear. Participants with NH had PTA < 20 dB HL in both ears. All subjects underwent audiometry, cognitive testing for IQ scores, language testing, and MRI scanning. Imaging data from 12 children had to be excluded from analysis, due to movement artifact (*n* = 1), inability to lie still in the scanner (*n* = 1), inadequate data acquisition (*n* = 7) and inadequate data due to the presence of metallic devices (orthodontic appliances, *n* = 2, and a bone-anchored hearing aid, *n* = 1). Thus, the analysis included 29 children with UHL (13 right UHL, 16 left UHL) and 20 siblings with NH. Children with both acquired and congenital UHL were included. All subjects were cognitively normal per parent report.

This study protocol was approved by the Human Research Protections Office (HRPO) at the Washington University School of Medicine. Informed consent was obtained from all parents of subjects and pediatric assent obtained from all minor participants.

### Baseline variables

Parents provided demographic, health, and family data. Parents also indicated whether children required speech therapy, special educational accommodations or special services at school, or an individualized educational program (IEP).

### Measured outcome variables

Cognitive ability was assessed by the Wechsler Abbreviated Scale of Intelligence (WASI) (Wechsler, 1999). We used standardized performance IQ, verbal IQ, and full scale IQ scores along with vocabulary T-scores as educational outcomes. All scores were standardized to a mean of 100 with a standard deviation (SD) of 15, except for the vocabulary T-score, which is standardized to a mean of 50 and SD of 10. Two standardized language tests were administered to the participants—the Oral and Written Language Scale (OWLS) and the Clinical Evaluation of Language Fundamentals (CELF) (Carrow-Woolfolk, 1995; Semel et al., 2011). Because both language tests use a standardized scale, they were combined as language outcomes for analysis.

### Mri scanning protocol and image acquisition

Images were obtained on a 3.0 Tesla Siemens Trio, MR scanner (Erlangen, Germany). The imaging protocol included T1-weighted Magnetization Prepared Rapid Acquisition Gradient Echo (MPRAGE) sequence [Time of repetition (TR) /inversion time (TI)/ echo time (TE) = 2400/1000/3.16 ms, voxel size = 1 × 1 × 1 mm^3^] and a T2-weighted (T2W) fast spin echo (FSE) scan (TR = 4380 ms, TE = 94 ms, 1 × 1 × 4 mm). DTI data (1.5 × 1.5 × 1.5 mm voxels, TR = 9600 ms, TE = 95 ms), was collected in 25 different directions with *b*-values linearly distributed between 0 and 1400 s/mm^2^.

The images were preprocessed and transformed into modified (Talairach and Tournoux, 1988) space using the following methods. A 9-parameter rigid body alignment registered all frames in all runs and was used for motion correction of each subject. Resampling was done by a 3-dimensional cubic spline interpolation and transformed to a Talairach atlas space using a single common atlas derived from adult and child brains via a warping mechanism (Burgund et al., 2002). After the registration steps, the diffusion data was processed with locally written software using a log linear algorithm into DTI parameter data using the commonly used tensor model (Basser et al., 1994).

### Regions of interests

The 15 regions of interest selected for analysis are listed in Table 1 and fall into two groups. The first are regions that are known to be involved in auditory processing. The second group are non-auditory regions that are considered important white matter areas that are commonly sampled in DTI studies of the brain.

**Table 1:** **MNI coordinates for regions of interest (ROI) used in the study**.

	***x***	***y***	***z***	**Average size (mm^3^)**	**Shape**
**AUDITORY ROIs**					
Auditory radiation, left middle	−32	−23	−5	96	Punctate linear
Auditory radiation, right	27	−23	−8	96	Punctate linear
Heschl's gyrus, gray matter left	−41	−21	8	25	Punctate arc
Heschl's gyrus, gray matter right	41	−20	5	23	Punctate arc
Heschl's gyrus, subcortical white matter left	−45	−20	7	22	Oblong
Heschl's gyrus, subcortical white matter right	44	−20	4	20	Oblong
Inferior colliculus, left	−6	−36	−8	26	Oblong
Inferior colliculus, right	2	−35	−8	27	Oblong
Lateral lemniscus, left	−9	−30	−8	34	Oblong
Lateral lemniscus, right	6	−29	−9	50	Oblong
Superior temporal gyrus, left	−68	−42	2	36	Crescent
Superior temporal gyrus, right	66	−41	7	45	Crescent
**CONTROL ROIs**					
Genu of corpus callosum	−1	21	15	122	Oblong
Splenium of corpus callosum	1	−34	18	188	Oblong
Middle cerebellar peduncle, left	−24	−40	−35	354	Oblong
Middle cerebellar peduncle, right	17	−40	−36	420	Oblong
Globus pallidus, left	−18	1	−24	147	Oblong
Globus pallidus, right	16	1	−3	174	Oblong
Putamen, left	−23	13	−3	147	Oblong
Putamen, right	20	13	−4	147	Oblong
Anterior corona radiata, left	−18	42	−7	122	Oblong
Anterior corona radiata, right	18	44	−8	150	Oblong
Anterior limb of the internal capsule, left	−14	7	6	90	Enlongated oblong
Anterior limb of the internal capsule, right	15	10	5	122	Enlongated oblong
Uncinate fasciculus, left	−20	21	−11	85	Oblong
Uncinate fasciculus, right	16	22	−12	91	Oblong
Inferior longitudinal fasciculus, left	−41	−31	−8	123	Oblong
Inferior longitudinal fasciculus, right	39	−31	−11	170	Oblong

*Average size was measured as mm^*3*^*.

**Figure 1:**
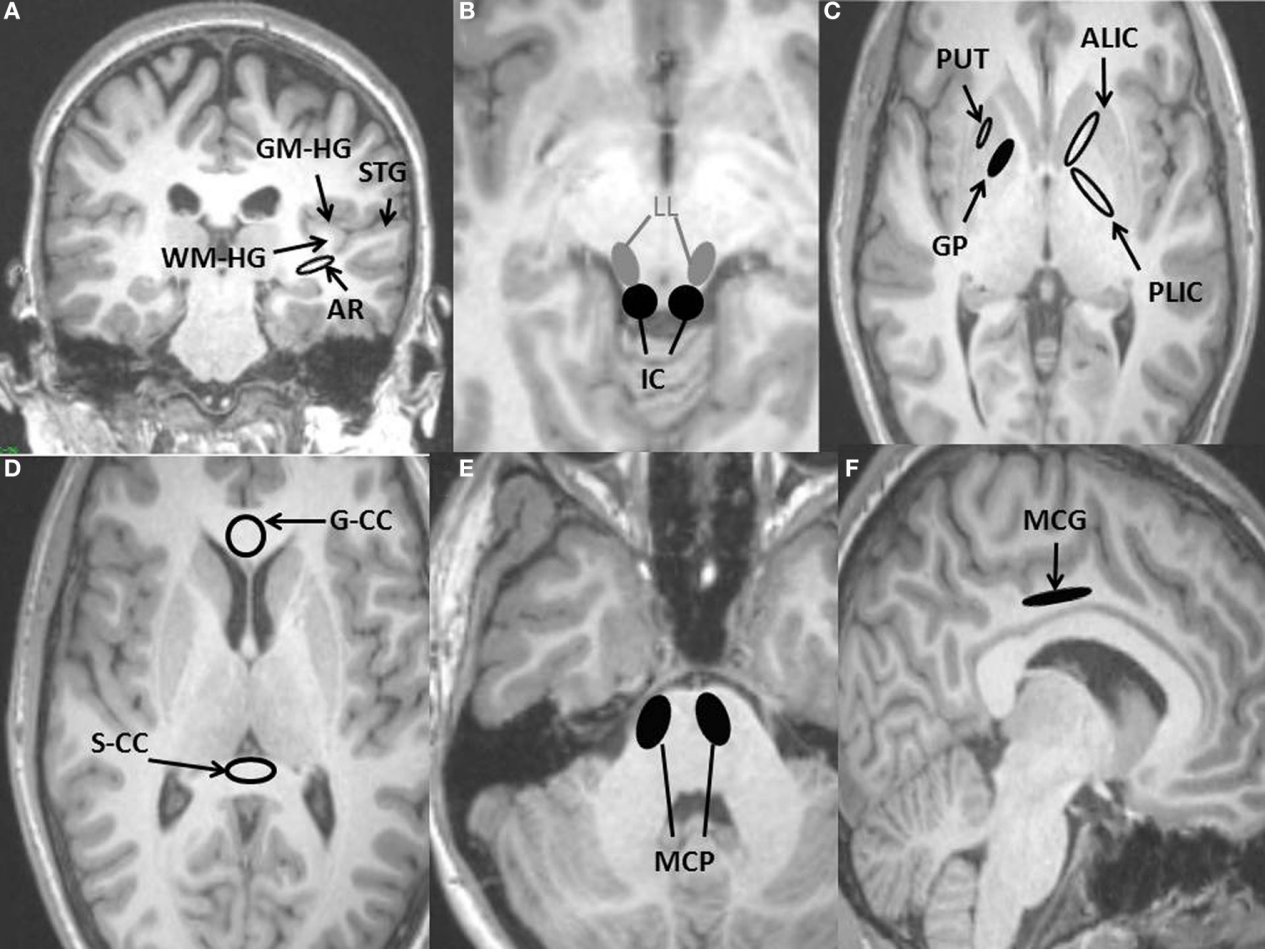
**Regions of interest (ROIs) used for DTI measurements placed on anatomical T1-weighted MRI scan. (A)** Four auditory ROIs, Gray matter of Heschl's gyrus (GM-HG), White matter of Heschl's gyrus (WM-HG), Superior temporal gyrus (STG), and Auditory radiation (AR). **(B)** Two auditory ROIs, Inferior colliculus (IC), Lateral lemniscus (LL). **(C)** Three non-auditory ROIs, Putamen (PUT), Globus pallidus (GP), Posterior limb of the internal capsule (PLIC), Anterior limb of internal capsule (ALIC). **(D)** Two non-auditory ROIs, Genu of the corpus callosum (G-CC), Splenium of the corpus callosum (S-CC). **(E)** Non-auditory ROI, Middle cerebellar peduncle (MCP). **(F)** Non-auditory ROI, Middle cingulate gyrus (MCG).

Auditory regions (Figures 1A,B) include: Auditory radiation; Heschl's gyrus (both gray and subcortical white matter); Inferior colliculus; Lateral lemniscus; Superior temporal gyrus (gray matter only).

**Figure 2:**
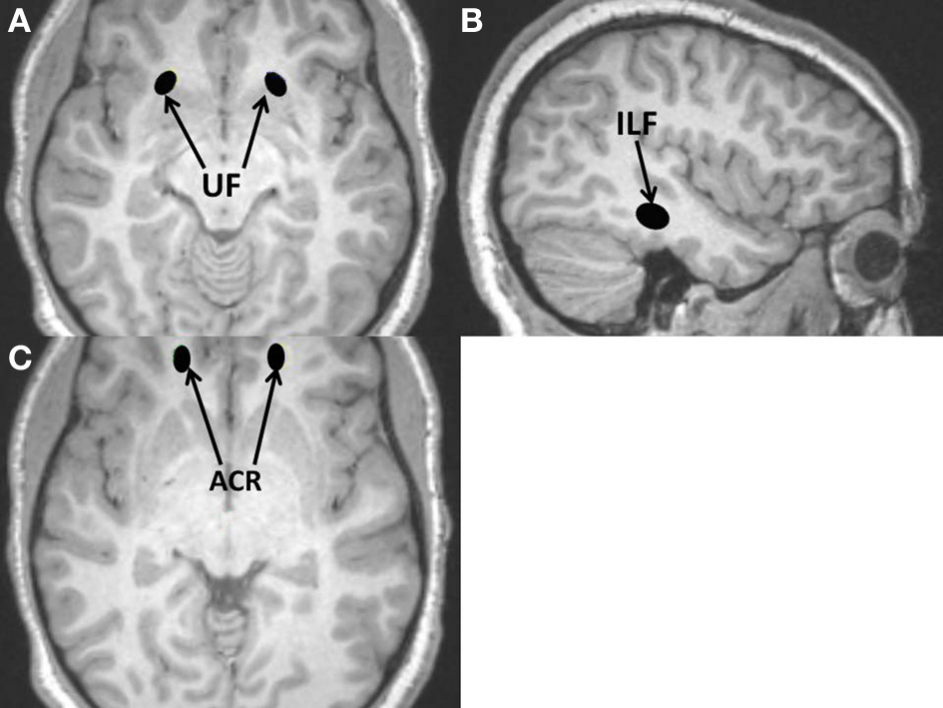
**Additional regions of interest (ROIs) used for DTI measurements placed on anatomical T1-weighted MRI scan; all are non-auditory ROIs. (A)** Uncinate fasciculus (UF); **(B)** Inferior longitudinal fasciculus (ILF); **(C)** Anterior corona radiata (ACR).

Non-auditory regions (Figures 1C–F, 2A–C) include: Genu and splenium of the corpus callosum; Middle cerebellar peduncle; Globus pallidus; Putamen; Anterior corona radiata; Anterior limb of the internal capsule; Uncinate fasciculus; and Inferior longitudinal fasciculus. These regions are commonly sampled in DTI studies due to their importance in brain function and chosen as “control” regions where no differences would be expected. The latter four were also chosen due to their involvement in executive functioning (Cummings, 1993; Schmahmann et al., 2008).

Regions were located and sampled after the MPRAGE scans were transformed into Tailarach space over three to four slices each by one independent rater under the supervision of an experienced neuroradiologist. ROI location was traced on the MPRAGE anatomy, but guided using coregistered T2-weighted images to avoid cerebrospinal fluid, and FA images to capture fiber centers. The FA and MD values in the ROIs were measured and averaged over consecutive slices for a more accurate measurement than a single slice would give, thus minimizing the risk of type I error. The approximate MNI coordinates for these ROIs are listed in Table 1 (each subject's regions are slightly different).

### Statistical analysis

Statistical analyses were performed using the Statistical Package for the Social Sciences (SPSS), Version 20.0 (IBM Corp., Armonk, NY, US). Educational performance variables were compared with Mann-Whitney *U*-tests between the UHL and NH groups and between the right and left UHL groups. Pearson's chi-square was used to assess need for speech therapy and/or IEP. We used a Bonferroni correction of 0.05/6 = 0.008 for a two-sided *alpha* threshold for the six educational outcomes assessed; a *p*-value less than 0.05 was considered a trend.

Kruskal-Wallis tests provided between-group comparisons of DTI parameters. Paired Wilcoxon signed rank-test of DTI parameters between sides (right and left) were performed within each of the groups. Correlations between educational variables and DTI parameters were explored using Spearman's rho coefficient. Due to the concerns of multiple comparisons, we used a Bonferroni correction of 0.05/12 = 0.004 for a two-sided *alpha* threshold for auditory ROIs (6 ROIs × 2 hemispheres), and 0.05/18 = 0.003 for non-auditory ROIs (9 ROIs × 2 hemispheres); a *p*-value less than 0.05 was considered a trend.

Because multiple variables can predict outcomes, we used multiple linear or logistic regression modeling to evaluate the role of age and UHL simultaneously with the specific DTI parameters which were statistically significant or trending toward significance in bivariate analysis on the educational outcomes of interest. For the regression analyses, a *p*-value less than 0.05 was considered statistically significant.

## Results

Demographic data is presented in Table 2. The age of study participants ranged from 7.4 to 17.6 years. There were no significant differences in gender, age, racial composition, handedness or rates of prematurity between the NH and UHL groups. Etiology of hearing loss did not differ significantly between the right UHL and left UHL groups. Age at identification of hearing loss was 3 months to 8 years with a mean of 4.6 years (*SD* 2.8 years); mean duration of UHL was 7.5 years (*SD* 3.3 years), with a range of 2.2–14.2 years.

**Table 2:** **Demographic characteristics of normal hearing (NH) children and children with unilateral hearing loss (UHL)**.

**Characteristic**	**NH (*n* = 20)**	**UHL (*n* = 29)**	***p*-value**	**Right UHL (*n* = 13)**	**Left UHL (*n* = 16)**	***P*-value**
Male sex, *n* (%)	14 (70)	13 (44.8)	0.082	6 (46.2)	3 (43.8)	0.897
Age in years, mean (*SD*)	12.7 (2.93)	12.2 (2.35)	0.514	11.8 (2.68)	12.5 (2.08)	0.430
Race, *n* (%)			0.805			0.970
Black	3 (15)	6 (20.7)		3 (23.1)	3 (18.8)	
White	15 (75)	18 (62.1)		8 (61.5)	10 (62.5)	
Asian	1 (5.0)	2 (6.9)		1 (7.7)	1 (6.2)	
Other	1 (5.0)	3 (10.3)		1 (7.7)	2 (12.5)	
Ethnicity, *n* (%)			0.401			0.359
Hispanic/latino	0 (0)	1 (3.4)		0 (0)	1 (6.2)	
Not hispanic or latino	20 (100)	28 (96.6)		13 (100)	15 (93.8)	
Handedness, *n* (%)			0.168			0.879
Right-handed	15 (75)	27 (93.1)		12 (92.3)	15 (93.8)	
Left-handed	4 (20.0)	2 (6.9)		1 (7.7)	1 (6.2)	
Both	1 (5.0)	0 (0)		0 (0)	0 (0)	
Hearing loss etiology						
Otitis media	–	2 (6.9)	–	0 (0)	2 (12.5)	0.186
Trauma	–	2 (6.9)	–	1 (7.7)	1 (6.2)	0.879
Meningitis	–	1 (3.4)	–	0 (0)	1 (6.2)	0.359
Congenital	–	5 (17.2)	–	2 (15.4)	3 (18.8)	0.569
Unknown	–	13 (44.8)	–	5 (38.5)	8 (50)	0.534
Other	–	4 (13.8)	–	3 (23.1)	1 (6.20	0.191
Prematurity	3 (15.0)	5 (17.2)	0.835	2 (15.4)	3 (18.8)	0.811

Though NH subjects performed better on all aspects of the cognitive testing, these differences were not all statistically significant (Table 3). No differences in full sum IQ were observed between the right and left UHL groups. However, NH individuals trended toward higher scores on the verbal component of the cognitive tests (Table 3). In addition, children with UHL had a significantly greater need for IEPs (45%) and speech therapy (41%) than children with NH (5% for both IEPs and speech therapy).

**Table 3:** **Comparisons between unilateral hearing loss (UHL) group and the normal hearing group (NH) for educational outcome variables, and right unilateral hearing loss (right UHL) and left unilateral hearing loss (left UHL) groups**.

**Educational outcome**	**UHL**	**NH control**	***p*-value**	**Right UHL**	**Left UHL**	***p-value***
**IQ SCORES, MEAN (*SD*)**						
Full sum	104 (24.5)	110 (14.9)	0.328	101 (23.1)	106 (26.2)	0.577
Performance	100 (18.2)	109 (15.7)	0.082	98.2 (24.7)	101 (11.1)	0.717
Verbal	100 (16.6)	109 (11.9)	0.046^*^	98 (18.3)	102 (15.4)	0.484
Vocabulary subscale	48 (12.7)	54 (7.4)	0.105	49 (13.9)	48 (12.0)	0.983
**NEED FOR SPECIAL SERVICES**						
Individualized educational program (*n*, %)	13 (44.8)	1 (5.0)	0.002^†^	5 (38.5)	8 (50)	0.534
Speech therapy (*n*, %)	12 (41.4)	1 (5.0)	0.005^†^	4 (30.8)	8 (50)	0.296

* Trend at p < 0.05;

† *Significant at p < 0.008 for multiple comparisons of educational outcomes*.

### Dti parameters between groups

DTI parameters were compared separately on the left and right side of the brain; for instance, the FA of the right auditory radiation of the UHL group vs. that of the NH group (Table 4). FA values trended toward being higher in the NH group than in the UHL group in two auditory regions and two non-auditory regions. The FA of the left lateral lemniscus was significantly higher for NH compared to UHL. The only trends between groups among mean diffusivities were in the MD of the in right subcortical white matter of Heschl's gyrus and left putamen. In general, FA was greater and MD lower in the NH group than in the UHL group, but this did not reach statistical significance in most regions.

**Table 4:** **DTI parameters in those with unilateral hearing loss (UHL; *n* = 29) and normal hearing (NH; *n* = 20) participants amongst six auditory regions of interest (ROI) and nine non-auditory ROIs**.

	**UHL**	**NH**	***p*-value**	**Right UHL**	**Left UHL**	***p*-value**
**AUDITORY ROIs**						
MD subcortical white matter of Heschl's gyrus, right	0.637	0.591	0.048^*^	0.643	0.632	0.130
FA lateral lemniscus, left	0.364	0.446	0.001^†^	0.381	0.351	0.005^*^
FA subcortical white matter of Heschl's gyrus, left	0.338	0.397	0.013^*^	0.365	0.315	0.009^*^
FA lateral lemniscus, right	0.392	0.457	0.025^*^	0.397	0.351	0.079
**NON-AUDITORY ROIs**						
MD Putamen, left	0.708	0.686	0.034^*^	0.715	0.703	0.081
FA anterior limb of the internal capsule, left	0.575	0.607	0.030^*^	0.571	0.578	0.093
FA centrum semiovale, right	0.468	0.503	0.015^*^	0.456	0.477	0.030^*^

*Additional columns list values for right unilateral hearing loss (Right UHL; n = 13) and left unilateral hearing loss (Left UHL; n = 16); adjacent p-value column refers to p-values of Kruskal-Wallis tests between right UHL, left UHL and normal hearing (NH). Only parameters with uncorrected p-values < 0.05 are listed*.

* Trend at p < 0.05 level;

† *Significant at p < 0.004 for auditory ROIs and <0.003 for non-auditory ROIs*.

We also compared DTI parameters of the right UHL and left UHL groups (Table 4). The FA of the left lateral lemniscus and the FA of the left subcortical white matter of Heschl's gyrus trended toward being lower in the left UHL group. The FA of the right centrum semiovale trended toward being higher in the left UHL group.

### Dti parameters between sides

Paired comparisons between the left and right sides of structures were performed for the UHL and NH groups. In all UHL subjects, the right side FA was greater than the left side FA in both auditory and non-auditory regions, with the exception of the superior temporal gyrus, middle cingulate gyrus, anterior limb of the internal capsule, centrum semiovale and the anterior corona radiata (Table 5). Amongst NH subjects, a similar pattern was observed with a few exceptions—superior temporal gyrus, Heschl's gyrus, anterior limb of the internal capsule, middle cingulate gyrus, and the anterior corona radiata. MD results generally corresponded to FA results but in the opposite direction. For both NH and UHL subjects, right sided MD was significantly lower than left sided MD in most structures.

**Table 5:** **Comparison of fractional anisotropy (FA) and mean diffusivity (MD) between right and left hemispheres for unilateral hearing loss (UHL; *n* = 29) and normal hearing (NH; *n* = 20) participants in six auditory and nine non-auditory regions of interest (ROI)**.

	**UHL**			**NH**		
	**Right FA mean**	**Left FA mean**	***p*-value**	**Right FA mean**	**Left FA mean**	***p*-value**
**AUDITORY ROI**						
Auditory radiation	0.536	0.490	<0.001^†^	0.539	0.501	0.037^*^
Heschl's gyrus	0.187	0.172	0.005^*^	0.200	0.193	0.296
Subcortical white matter of Heschl's gyrus	0.393	0.338	0.004^†^	0.449	0.397	<0.001^†^
**NON-AUDITORY ROI**						
Posterior limb of the internal capsule	0.704	0.635	<0.001^†^	0.712	0.641	<0.001^†^
Middle cerebellar Peduncle	0.691	0.622	<0.001^†^	0.701	0.646	0.004^*^
Uncinate fasciculus	0.482	0.431	<0.001^†^	0.481	0.459	0.135
Middle cingulate gyrus	0.612	0.637	0.071	0.649	0.674	0.025^*^
Putamen	0.186	0.181	0.364	0.206	0.181	<0.001^†^
	**Right MD mean**	**Left MD mean**	***p*-value**	**Right MD mean**	**Left MD mean**	***p*-value**
**AUDITORY ROI**						
Auditory radiation	0.608	0.667	<0.001^†^	0.614	0.654	0.040^*^
Heschl's gyrus	0.738	0.777	0.001^†^	0.709	0.766	<0.001^†^
Inferior colliculus	0.728	0.740	0.552	0.657	0.696	0.033^*^
Lateral lemniscus	0.668	0.724	0.008^*^	0.617	0.684	0.009^†^
Subcortical white matter of Heschl's gyrus	0.637	0.722	<0.001^†^	0.591	0.707	<0.001^†^
**NON-AUDITORY ROI**						
Posterior limb of the internal capsule	0.607	0.673	<0.001^†^	0.594	0.667	<0.001^†^
Middle cerebellar peduncle	0.595	0.549	0.001^†^	0.587	0.536	0.001^†^
Anterior limb of the internal capsule	0.592	0.668	<0.001^†^	0.579	0.656	<0.001^†^
Globus pallidus	0.594	0.616	0.020^*^	0.571	0.594	0.057
Putamen	0.684	0.708	<0.001^†^	0.676	0.686	0.093
Centrum semiovale	0.655	0.686	0.001^†^	0.639	0.671	<0.001^†^
Inferior longitudinal Fasciculus	0.690	0.714	0.039^*^	0.674	0.698	0.232

*Only parameters with uncorrected p-values < 0.05 are listed*.

* *Trend at p < 0.05 level*;

† Significant at p < 0.004 for auditory ROIs and <0.003 for non-auditory ROIs.

### Correlations

Correlations of educational outcome variables with DTI parameters were performed separately for the following groups: (1) All NH subjects; (2) All UHL subjects; (3) All Right UHL subjects; and (4) All Left UHL subjects.

In general, FA correlated positively and MD negatively with test scores for children with UHL (data not shown). Though there were many trends toward significance in the NH group, there were no significant correlations. In the UHL group, the FA and the MD of several ROIs correlated significantly with several different educational parameters, such as full IQ, verbal IQ, IEP, and speech therapy (Table 6). The FA of the left uncinate fasciculus was negatively correlated with Performance and Full IQ, the MD of the right posterior limb of the internal capsule was negatively associated with Verbal IQ, while the FA of the left middle cerebellar peduncle was associated with need for speech therapy. For children with right UHL, the MD of the right middle cerebellar peduncle was highly correlated with Verbal IQ, and the FA of both right and left uncinate fasciculus were highly correlated with both Performance and Full IQ. For children with left UHL, both the MD and FA of the left middle cerebellar peduncle were strongly correlated with speech therapy.

**Table 6:** **Spearman rho correlation values between educational outcomes and DTI parameters (fractional anisotropy, FA; mean diffusivity, MD) in auditory and non-auditory regions of interest (ROI) in participants with normal hearing (NH), unilateral hearing loss (UHL), right UHL or left UHL**.

	**Performance IQ**	**Full IQ**	**Verbal IQ**	**Vocabulary T-score**	**IEP**	**Speech-language therapy**	**Language standard score**
**AUDITORY ROIs**							
**NH (*n* = 20)**							
No statistically significant correlations							
**UHL (*n* = 29)**							
FA left Heschl's gyrus	−0.159	0.047	0.367	0.355	−0.522^†^	−0.167	0.262
**Right UHL only (*n* = 13)**							
No statistically significant correlations							
**Left UHL only (*n* = 16)**							
No statistically significant correlations							
**NON−AUDITORY ROIs**							
**NH (*n* = 20)**							
No statistically significant correlations							
**UHL (*n* = 29)**							
MD right posterior limb of the internal capsule	−0.038	−0.171	−0.485^†^	−0.435^*^	0.448^*^	0.343	−0.462^*^
FA left middle cerebellar peduncle	−0.072	0.275	0.474^*^	0.342	−0.324	−0.632^†^	0.270
FA left uncinate fasciculus	−0.569^†^	−0.554^†^	−0.358	−0.406^*^	0.265	0.192	−0.438^*^
FA left middle cingulate gyrus	0.056	0.156	0.223	0.150	−0.075	−0.460^*^	0.130
**Right UHL only (*n* = 13)**							
MD right middle cerebellar peduncle	−0.595	−0.086	−0.905^†^	−0.795^*^	0.252	0.504	−0.738^*^
FA left uncinate fasciculus	−0.819^†^	−0.841^††^	−0.451	−0.338	−0.254	0.178	−0.576^*^
FA right uncinate fasciculus	−0.720^†^	−0.725^†^	−0.566^*^	−0.524	0.380	0.579	−0.736^†^
**Left UHL only (*n* = 16)**							
MD left middle cerebellar peduncle	0.147	−0.377	−0.430	−0.328	0.412	0.866^††^	−0.355
FA left middle cerebellar peduncle	−0.019	0.580^*^	0.606^*^	0.455	−0.454	−0.784^†^	0.517

Statistical significance was considered to be p < 0.004 for auditory ROIs and p < 0.003 for non-auditory ROIs.

* p < 0.05;

† p < 0.01;

†† p < 0.001.

IQ, intelligence quotient; IEP, individualized education plan.

### Regression analyses

As DTI parameters have been noted to change with age (Loenneker et al., 2011), we performed multiple linear regression analyses for regions with significant correlations with educational outcome variables as the dependent variable; age, hearing loss status and one DTI parameter (either FA or MD) served as independent variables. As with the correlation analyses, FAs were generally positively related to test scores while MDs were negatively associated with test scores (Table 7).

**Table 7:** **Results of multiple linear regression analyses for all subjects (*n* = 49)**.

**Outcome**	**Unstandardized coefficient**	**Standard error**	***t*-value**	***p*-value**
**LANGUAGE COMPOSITE SCORE**				
Intercept	−3.79	46.14	−0.08	0.94
MD left Putamen	133.0	61.05	2.18	0.035
Age	1.65	0.84	1.96	0.056
UHL	−15.70	4.40	−3.57	0.001
Intercept	61.23	18.81	3.26	0.002
FA right inferior longitudinal fasciculus	55.75	26.24	2.13	0.039
Age	1.12	0.81	1.38	0.175
UHL	−11.66	4.27	−2.73	0.009
**FULL IQ**				
Intercept	43.56	32.39	1.35	0.188
FA right middle cerebellar peduncle	80.44	35.26	2.28	0.029
Age	0.76	1.33	0.57	0.57
UHL	−7.59	7.01	−1.08	0.286
**PERFORMANCE IQ**				
Intercept	184.2	22.87	8.05	<0.001
FA left uncinate fasciculus	−129.0	46.54	−2.77	0.008
Age	−1.30	0.89	−1.45	0.153
UHL	−13.20	4.76	−2.77	0.008
**VERBAL IQ**				
Intercept	44.47	24.20	1.84	0.073
FA splenium of the corpus callosum	62.67	24.03	2.61	0.012
Age	1.14	0.82	1.40	0.168
UHL	−7.90	4.12	−1.92	0.061
Intercept	140.5	21.10	6.66	<0.001
MD right middle cerebellar peduncle	−78.73	30.13	−2.61	0.013
Age	1.16	0.87	1.34	0.189
UHL	−10.24	4.60	−2.23	0.033
Intercept	50.93	20.67	2.47	0.019
FA left middle cerebellar peduncle	61.70	24.21	2.55	0.016
Age	1.43	0.88	1.64	0.112
UHL	−9.31	4.65	−2.00	0.054

Standardized scores for educational outcomes were the dependent variables, and the DTI parameters (FA, Fractional Anisotropy; MD, Mean Diffusivity) for the stated regions of interest, age and hearing status (i.e., unilateral hearing loss, UHL) were entered in the models as predictors. FA, Fractional Anisotropy; MD, Mean Diffusivity.

Multiple logistic regression analyses were performed similarly for IEP and speech therapy as the dependent variables. The FAs of the bilateral middle cerebellar peduncles were negatively related to the need for speech therapy (Table 8). Hearing loss status was associated with educational outcome variables in several models.

**Table 8:** **Results of multiple logistic regression analyses for all subjects (*n* = 49)**.

	**Unstandardized coefficient**	**Standard error**	**Wald statistic**	***p*-value**
**SPEECH THERAPY**				
Constant	12.17	4.90	6.17	0.013^*^
FA left middle cingulate gyrus	−15.16	7.07	4.60	0.032^*^
Age	−0.05	0.16	0.11	0.740
UHL	−2.28	1.14	4.03	0.045
Constant	−11.75	7.13	2.72	0.099
MD left middle cerebellar peduncle	28.64	12.60	5.16	0.023^*^
Age	−0.19	0.21	0.80	0.372
UHL	−2.39	1.30	3.40	0.065
Constant	14.30	5.66	6.38	0.012^*^
FA left middle cerebellar peduncle	−15.51	6.44	5.80	0.016^*^
Age	−0.22	0.19	1.37	0.242
UHL	−2.49	1.29	3.70	0.054
Constant	13.78	6.23	4.89	0.027^*^
FA right middle cerebellar peduncle	−12.44	6.02	4.27	0.039^*^
Age	−0.24	0.20	1.50	0.220
UHL	−2.68	1.29	4.29	0.038^*^
**INDIVIDUALIZED EDUCATIONAL PROGRAM**				
Constant	−15.94	10.35	2.37	0.124
MD left Heschl's gyrus	30.07	14.14	4.52	0.033^*^
Age	−0.37	0.19	4.01	0.045^*^
UHL	−3.13	1.23	6.47	0.011^*^
Constant	−30.15	14.4	4.38	0.036^*^
MD left superior temporal gyrus	46.09	18.62	6.13	0.013^*^
Age	−0.10	0.21	0.22	0.641
UHL	−4.59	1.72	7.13	0.008^†^
Constant	13.70	4.55	9.05	0.003^†^
FA left Heschl's gyrus	−43.75	19.15	5.22	0.022^*^
Age	−0.24	0.19	1.61	0.204
UHL	−3.55	1.40	6.45	0.011^*^
Constant	17.65	5.83	9.16	0.002^†^
FA left superior temporal gyrus	−52.17	20.89	6.24	0.013^*^
Age	−0.45	0.21	4.64	0.031^*^
UHL	−3.49	1.36	6.57	0.01^*^
Constant	−28.51	14.52	3.85	0.050
MD left posterior limb of the internal capsule	51.94	21.27	5.97	0.015^*^
Age	−0.24	0.20	1.51	0.219
UHL	−3.79	1.43	7.06	0.008^†^
Constant	−10.73	8.19	1.72	0.190
MD right posterior limb of the internal capsule	27.94	12.79	4.77	0.029^*^
Age	−0.29	0.19	2.38	0.123
UHL	−2.99	1.21	6.09	0.014^*^

Standardized scores for educational outcomes were the dependent variables, and the DTI parameters (FA, Fractional Anisotropy; MD, Mean Diffusivity) for the stated regions of interest, age and hearing status (i.e., unilateral hearing loss, UHL) were entered in the models as predictors.

* p < 0.05;

† p < 0.01.

## Discussion

Our study is one of the first to apply DTI analysis to children with UHL. Consistent with previous research, children with UHL had significantly worse speech and language scores and required speech therapy and IEPs more often than children with NH (Lieu et al., 2010). Some significant differences were found between DTI parameters of the NH and UHL groups, or between right UHL, left UHL, and NH groups of children. Left-right asymmetries noted in the NH children were retained in the children with UHL.

The main advantage and the most interesting results from our study come from the inclusion of multiple educational outcome variables. DTI parameters in several ROIs were significantly correlated with educational outcome variables. Interestingly, the greater the FA of left Heschl's gyrus, the less likely a child needed an IEP, indicating that the greater the organization in this region, the better the educational outcome (Tables 6, 8). Notably, IEPs are provided through public schools only when children are diagnosed with an educationally significant problem, such as reading delays or behavioral problems; speech delays are only one diagnosis that would elicit an IEP. In contrast, speech therapy may be pursued privately, especially when a child attends a private school. Heschl's gyrus is tonotopically organized, and monaural deprivation disrupts this organization (Popescu and Polley, 2010). Although this area is anatomically crucial in auditory functioning, the association between test scores and the microstructure of this region requires further confirmation and validation in future studies. As neuroimaging is often an early step in the etiologic work-up of pediatric UHL, acquisition of DTI would be a feasible addition to the protocol and may provide clinically relevant information in regard to special educational needs.

Proposed by Kimura (1963), the dichotic listening paradigm held that the right ear was preferred for listening to speech, due to the predominant representation of a right ear stimulus in the left cerebral hemisphere, where language typically lateralizes (Kimura, 1963). According to a hypothesis of right ear advantage, children with left UHL should enjoy a speech-language advantage, but the evidence is currently inconclusive (Hartvig et al., 1989; Niedzielski et al., 2006; Lieu et al., 2010). The only DTI differences observed between right and left UHL were increased FAs for right UHL in the left lateral lemniscus and the subcortical white matter of the left Heschl's gyrus. Instead, several DTI regions showed differential strengths in correlation with language and verbal IQ outcomes in children with and without UHL. These discordances suggest that the brains of children with UHL undergo reorganization in the white matter to help compensate for the lack of typical peripheral auditory stimuli to the contralateral hemisphere. Thus, the dichotic listening paradigm may not fit when one ear does not hear.

Regardless of hearing status, rightward asymmetries were observed in FA in many auditory structures. In non-auditory structures, asymmetries in DTI parameters were mixed, making interpretation of our results in large structures such as the centrum semiovale and posterior limb of the internal capsule less clear (Bonekamp et al., 2007; Eluvathingal et al., 2007; Liu et al., 2010; Takao et al., 2011). In auditory structures, our findings indicate that despite lack of auditory input from one ear, patterns of microstructural integrity are preserved in children with UHL. These results contrast with stimuli-induced functional MRI findings. When sounds or speech are presented monaurally to a NH individual, there is increased activation in the contralateral primary auditory cortex (Scheffler et al., 1998). Adults with UHL have a more symmetrical fMRI activation, with decreased contralateral primary auditory cortex activation and increased ipsilateral primary auditory cortex activation, when presented with a speech stimulus to the hearing ear (Firszt et al., 2006). While we would have expected more white matter microstructural differences in auditory structures between children with and without UHL, the lack thereof may be due to compensatory plasticity in the face of monaural deprivation, with these structures transmitting signal from the good ear, or being recruited for other brain functions. It is unclear whether UHL affects attentional networks and other aspects of executive functioning. In another study looking at fMRI data in children with and without UHL, children with UHL were found to have less activation of secondary auditory areas compared to NH individuals and failure to activate attentional areas (Propst et al., 2010). In the present cohort, differences in rs-fcMRI were found in areas associated with auditory processing, executive function and memory formation between children with UHL and NH controls (Tibbetts et al., 2011). Since the neuroanatomical microstructure remains intact, it is possible that these underutilized auditory areas have been recruited by other systems in the brain, such as noted by Obretenova et al. (2010), or analogously as has been shown to occur in the occipital cortex of blind subjects (Burton et al., 2012). An early-deaf and early-blind individual who relied on tactile communication modalities was found to have enhanced occipital connectivity as well greater activation of superior temporal and inferior frontal language regions on fMRI relative to a normally sighted and hearing person. This hypothesis could explain the discrepancy in speech-language outcomes between children with and without UHL despite the preservation of the microstructure in auditory regions. In contrast to our results here, early blind individuals were noted to have marked differences in the geniculocalcarine tract from normally sighted individuals; however, connections between the visual cortex and the orbital frontal and temporal cortices were preserved in early blind individuals (Shimony et al., 2006).

Contrary to expectations of finding differences in DTI parameters of auditory regions between children with and without UHL, most DTI parameters between the two groups were quite similar. Thus, given the results from prior studies on bilateral hearing loss, UHL seems to affect white matter development differently than bilateral hearing loss. There were no differences between left and right UHL groups. However, our sample size was small, so that our negative findings do not preclude the existence of such differences. We included children with both acquired and congenital hearing loss, which may have influenced our results, although in all cases the hearing loss was at birth or in early childhood. Because most of the children were born before the era of newborn hearing screening, precise onset of UHL could not be determined. However, the severity of UHL and universal lack of patient complaint about hearing indicates that the children were very young and unaware of the hearing loss when it occurred. Sampling ROIs manually may also be perceived as a limitation of this study; however, this is how many of the comparison studies were done, and given the small size of the structures we were sampling, alternative approaches, such as atlas-based approaches, were not feasible.

A few studies have used DTI to examine white matter microstructure in adults with hearing loss. There is a body of evidence indicating that adults with hearing loss have reduced FA in several structures. Chang et al. (2004) examined five ROIs (the lateral lemniscus and inferior colliculus, the trapezoid body, auditory radiation and superior olivary nucleus); reduced FA was observed in subjects with sensorineural hearing loss at all locations bilaterally with the exception of the trapezoid body (Chang et al., 2004). A study of 13 adults with early deafness revealed decreased FA within the temporal white matter, internal capsule, superior longitudinal fasciculus and the inferior frontal white matter (Kim et al., 2009). In adults with UHL, FA values of the inferior colliculus and the lateral lemniscus were significantly lower on the side contralateral to the hearing loss than those on the ipsilateral side (Lin et al., 2008). The study of Wu et al. looked at 12 subjects with unilateral congenital cochlear nerve deficiency and noted bilateral decrease in FA and increase in MD in the lateral lemniscus and inferior colliculus (Wu et al., 2009). However, the population of this study spanned a wide age distribution (age 8–29 years) and no correction was made for this wide variability in age. Unlike the results of previous studies, our findings indicate that microstructural integrity in children with UHL is not substantially altered from that of children with NH. The difference in our results could be related to the differences in our cohorts (unilateral vs. bilateral hearing loss in most other studies), to the age of our groups (children vs. adults, or mixed in other studies), and to our statistical methods.

Two studies have used DTI in children with bilateral prelingual deafness to investigate alternations in brain white matter tracts. Children (age 10–18 years) with bilateral prelingual deafness had lower FA values and increased radial diffusivity bilaterally in the superior temporal gyrus, Heschl's gyrus, planum polare, and splenium of the corpus callosum compared to controls with normal hearing (Miao et al., 2013). In addition, the mean radial diffusivity of the right superior temporal gyrus appeared to be correlated with the duration of sign language use. Chang and colleagues used DTI characteristics to compare children who had undergone cochlear implantation but were classified as having “good” or “poor” auditory performance outcomes (Chang et al., 2012). They found higher FA values in Broca's area, genu of the corpus callosum, and auditory tract among the children with “good” auditory performance compared to those with “poor” outcome. Strong correlations were found between FA and auditory performance scores with several brain areas: medial geniculate nucleus, Broca's area, genu of the corpus callosum, and auditory tract. They concluded that preoperative functional imaging prior to placement of cochlear implants might be helpful to understanding clinical outcomes. Although all of the brain areas from these two studies in children are different from the ones identified in adults with hearing loss, most but not all occurred in areas associated with auditory and language processing, and most showed bilateral changes. These findings in children with bilateral prelingual deafness are highly interesting, but they differ from the results of the current study. Whether they apply to the population of children with UHL who have preserved auditory stimulation in one ear would need further investigation.

## Conclusion

This study detected correlations between educational outcomes and microstructural integrity of brain structures in children with and without UHL that may have clinical relevance in the guidance of speech and language therapy. Our results imply that unilateral auditory input preserves many of the asymmetries in white matter microstructural patterns in children between right and left hemispheres. However, UHL results in functional and behavioral differences on language and educational measures, possibly due to recruitment of certain areas (e.g., middle cerebellar peduncle, superior temporal gyrus, as shown in Table 8) for other brain functions.

## Author contributions

Judith E. C. Lieu conceived and supervised the project and wrote the paper. Joshua S. Shimony supervised the project and wrote the paper. Tara Rachakonda collected data, performed statistical analyses, and wrote the paper. Rebecca S. Coalson collected data, performed statistical analysis, and revised the paper.

### Conflict of interest statement

The Associate Editor, Dr. Peelle Jonathan declares that, despite being affiliated to the same institution as authors The Department of Otolaryngology of Washington University School of Medicine, the review process was handled objectively. The rest of the authors declare that the research was conducted in the absence of any commercial or financial relationships that could be construed as a potential conflict of interest.
